# Evidence of somatic hypermutation in the antigen binding sites of patients with CLL harboring IGHV genes with 100% germline identity

**DOI:** 10.3389/fonc.2022.1079772

**Published:** 2022-12-14

**Authors:** Electra Sofou, Laura Zaragoza-Infante, Nikolaos Pechlivanis, Georgios Karakatsoulis, Sofia Notopoulou, Niki Stavroyianni, Fotis Psomopoulos, Elisavet Georgiou, Anne Langlois de Septenville, Frederic Davi, Andreas Agathangelidis, Anastasia Chatzidimitriou, Kostas Stamatopoulos

**Affiliations:** ^1^ Institute of Applied Biosciences, Centre for Research and Technology Hellas, Thessaloniki, Greece; ^2^ Laboratory of Biological Chemistry, School of Medicine, Faculty of Health Sciences, Aristotle University of Thessaloniki, Thessaloniki, Greece; ^3^ Hematology Department and HCT Unit, G. Papanicolaou Hospital, Thessaloniki, Greece; ^4^ Department of Hematology, APHP, Hôpital Pitié-Salpêtrière and Sorbonne University, Paris, France; ^5^ Department of Biology, School of Science, National and Kapodistrian University of Athens, Athens, Greece; ^6^ Department of Molecular Medicine and Surgery, Karolinska Institute, Stockholm, Sweden

**Keywords:** CLL (chronic Lymphocytic Leukemia), B cell receptor, antigen binding, somatic hypermutation, immunoglobulin genes

## Abstract

Classification of patients with chronic lymphocytic leukemia (CLL) based on the somatic hypermutation (SHM) status of the clonotypic immunoglobulin heavy variable (IGHV) gene has established predictive and prognostic relevance. The SHM status is assessed based on the number of mutations within the IG heavy variable domain sequence, albeit only over the rearranged IGHV gene excluding the variable heavy complementarity determining region 3 (VH CDR3). This may lead to an underestimation of the actual impact of SHM, in fact overlooking the most critical region for antigen-antibody interactions, i.e. the VH CDR3. Here we investigated whether SHM may be present within the VH CDR3 of cases bearing ‘truly unmutated’ IGHV genes (i.e. 100% germline identity across VH FR1-VH FR3) employing Next Generation Sequencing. We studied 16 patients bearing a ‘truly unmutated’ CLL clone assigned to stereotyped subsets #1 (n=12) and #6 (n=4). We report the existence of SHM within the germline-encoded 3’IGHV, IGHD, 5’IGHJ regions of the VH CDR3 in both the main IGHV-IGHD-IGHJ gene clonotype and its variants. Recurrent somatic mutations were identified between different patients of the same subset, supporting the notion that they represent true mutational events rather than technical artefacts; moreover, they were located adjacent to/within AID hotspots, pointing to SHM as the underlying mechanism. In conclusion, we provide immunogenetic evidence for intra-VH CDR3 variations, attributed to SHM, in CLL patients carrying ‘truly unmutated’ IGHV genes. Although the clinical implications of this observation remain to be defined, our findings offer a new perspective into the immunobiology of CLL, alluding to the operation of VH CDR3-restricted SHM in U-CLL.

## Introduction

The somatic hypermutation (SHM) status of the clonotypic, rearranged immunoglobulin heavy variable (IGHV) gene is a cornerstone for risk stratification of patients with chronic lymphocytic leukemia (CLL) (1–4). Depending on the SHM burden, i.e. the number of mutations within the sequence of the rearranged IGHV gene, cases are classified in two categories, namely unmutated CLL (U-CLL) and mutated CLL (M-CLL), with different biological background and clinical behavior (5–8).

The established approach for determining the SHM burden relies on the robust identification of nucleotide changes across the sequence of the rearranged IGHV gene, excluding the heavy variable complementarity determining region 3 (VH CDR3). This is mostly due to the difficulty in discriminating between actual SHM and random nucleotides added in the junction between the recombined IGHV, IGHD and IGHJ genes (9–11). However, this approach may result in the underestimation of the actual impact of SHM, in fact overlooking the most critical region for antigen-antibody interactions, i.e. the VH CDR3.

Here we investigated the possibility that SHM may also be present in CLL cases bearing ‘truly unmutated’ clonotypic IGHV genes (i.e. those with 100% germline identity across the VH FR1-VH FR3). To that end, we focused on two well characterized major stereotyped subsets: subset #1 (clan I IGHV genes/IGHD6-19/IGHJ4) and subset #6 (IGHV1-69/IGHD3-16/IGHJ3), displaying germline-encoded amino acid (aa) motifs QWL and YDYVWGSY within the respective VH CDR3 that originate from the IGHD6-19 and the IGHD3-16 gene, respectively (12). However, as reported by previous Sanger-based studies, patients assigned to both subsets can exhibit variations in these motifs that could potentially represent SHM events (12).

In order to address our starting question, we studied the IG gene repertoire of cases assigned to subsets #1 and #6 utilizing next generation sequencing (NGS), which enables the assessment of the subclonal architecture of antigen receptor gene repertoires at a high resolution (13, 14). We report VH CDR3-focused variations, very likely attributed to SHM, in CLL patients carrying ‘truly unmutated’ IGHV genes. While the small number of our cohort limits our capacity to safely predict the clinical relevance of this observation, our findings highlight the possible need to reappraise definitions regarding the characterization of the SHM status in CLL.

## Materials and methods

### Patient cohort

The study group comprised 12 patients assigned to the stereotyped subset #1 and 4 patients assigned to stereotyped subset #6. Detailed information on the immunogenetic features of the clonotypic IGHV-IGHD-IGHJ gene rearrangements is provided in **Supplemental Table 1**. All 16 patients were selected for exhibiting a ‘truly-unmutated’ IGHV status i.e. 100% germline IGHV identity as determined by Sanger sequencing. The study was approved by the local Ethics Review Boards of the participating institutions and was conducted in accordance with the declaration of Helsinki.

### IGHV-IGHD-IGHJ amplification and high-throughput sequencing methodology

Total RNA was isolated from Peripheral Blood Mononuclear Cells (PBMCs) and 1 μg was reverse transcribed to cDNA with the SuperScript™ II Reverse Transcriptase (Invitrogen, UK). IGHV-IGHD-IGHJ rearrangements were PCR amplified from 40 nanograms of cDNA using the Platinum™ Taq DNA Polymerase (Invitrogen, UK). IGHV subgroup-specific forward primers annealing to the leader region of the respective IGHV gene (depending on the case), and reverse primers annealing either to the IGHJ gene or the IGHC genes were utilized, the latter in order to amplify isotype-specific transcripts (15, 16). All amplicons were gel-purified with the Monarch^®^ DNA Gel Extraction Kit (New England Biolabs, USA) and 85 ng were used for library preparation. NGS libraries were prepared with a Dual Indexing sequencing approach according to the manufacturer’s instructions (NEBNext^®^ Ultra™ II FS DNA Library Prep Kit, New England Biolabs, USA). Library quantification was performed with Qubit™ (ThermoFisher Scientific), whereas purity and size were checked with the Fragment Analyzer™ Automated CE System (Agilent Technologies, USA). Paired-end NGS was carried out on a MiSeq Benchtop Sequencer (MiSeq reagent kit v3, 2x300 bp, Illumina Inc.)

### Bioinformatics analysis

Base calling, adapter trimming, and demultiplexing were performed by the Illumina signal processing software. Quality control of the raw NGS data was performed with a purpose-built, in-house pipeline. Briefly, we performed length and quality filtering, excluding low quality reads, then merged the paired-end NGS reads, followed again by length and quality filtering of the joined, full-sequence reads. Finally, to ensure high sequence quality in the VH CDR3 region, we included an additional filter that ensured a Qscore>=30 for nucleotides in the 30-45 nucleotide stretch ahead of the GXG motif in the FR4 region (**Supplemental Table 2**). High-quality sequences were then annotated with IMGT/HighV-QUEST (https://www.imgt.org) and meta-data analysis was performed with tripr (17) and custom scripts in R. To further ensure that biases due to sequencing errors would not be taken into account, we used the OLGEN coverage limit calculator (18) (http://app.olgen.cz/clc) in order to determine the minimum number of reads per variant that should be considered for analysis, based on the sequencing error induced during the NGS process. In our case, for a sequencing error of 2.4%, determined by the percentage of sequences aligned to PhiX, we set the minimum cutoff of reads at 66.

### Definitions

Clonotypes were defined as unique IGHV-IGHD-IGHJ nucleotide gene rearrangement sequences. The clonotype accounting for the majority of reads in a given sample was characterized as the main variant. All clonotypes utilizing the same IGHV gene and bearing a VH CDR3 amino acid sequence of same length as the main variant but exhibiting a maximal difference of up to two amino acids were defined as subclonal variants. Subclonal variants displaying different isotypes, namely IGHG or IGHA, were defined as switched variants.

### Statistical analysis and data visualization

Descriptive statistics for clonotype computation included counts and frequency distributions. For quantitative variables, we calculated the mean, median and minimum/maximum values. The Mann-Whitney U test was used to compare the levels of subclonal heterogeneity between subsets #1 and #6. The Kruskal-Wallis test was used to assess the presence of any amino acid positions in the VH CDR3 germline-encoded regions with significant differences in mutation frequencies. The Wilcoxon rank sum test was then used for *post-hoc* comparisons, in order to identify the exact amino acid positions for which the aforementioned significance was observed. P-values were adjusted using the Holm-Bonferroni correction. A significance level of α=0.05 was set. Data visualization was performed with custom R scripts.

## Results

### Overview of the NGS output

Forty PCR amplicons were sequenced, in particular: 36 amplicons corresponded to the mu, gamma and alpha transcripts of 12 cases assigned to subset #1, while 4 amplicons corresponded to mu transcripts of 4 cases assigned to subset #6.

In total, 8,955,886 raw reads were generated (median 215,546 reads/sample, range 109,639-466,729) corresponding to 5,855,795 merged, full-sequence reads (median 142,719 reads/sample, range 63,298-341,654), of which 5,329,188 (median 123,323/sample, range 55,282-320,999) represented productive IGHV-IGHD-IGHJ gene rearrangements. As the scope of the study was to examine the impact of SHM within the VH CDR3 region of ‘truly unmutated’ gene rearrangements, we excluded from downstream analysis any subclonal variants that exhibited nucleotide changes across the germline VH FR1 - VH FR3 part of the VH domain. Hence, we ended up with a total of 2,409,663 ‘truly-unmutated’ IGHV-IGHD-IGHJ gene rearrangement sequences (median 109 sequences/sample, range 33-198,699) as defined by their 100% identity to the respective IGHV germline gene.

### Clonal distribution and subclonal heterogeneity of ‘truly-unmutated’ IGHV-IGHD-IGHJ gene rearrangements in the BcR IG repertoire

**Figure 1:**
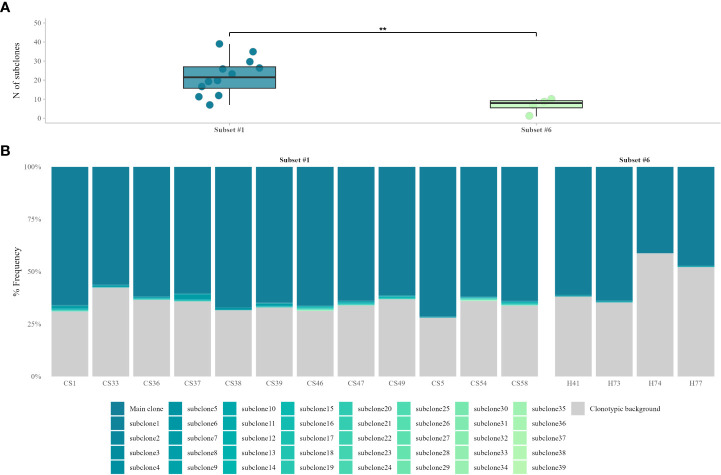
VH CDR3-derived, subclonal heterogeneity in ‘truly unmutated’ BcR IG rearrangements. **(A)** Comparison of subclonal branching of the VH CDR3 between the two stereotyped subsets shows significant differences (p = 0.005) in the number of subclonal variants, with subset #1 displaying higher VH CDR3 intraclonal variability. **(B)** Contribution of ‘truly unmutated’ IGHV-IGHD-IGHJ rearrangements to the total repertoire, for stereotyped subsets #1 and #6. The main CLL clonotype and its respective CLL-related, ‘truly unmutated’ subclonal variants are depicted in different shades of blue. The clonotypic background consists of either sequences bearing SHM in the rearranged IGHV gene, subclonal variants with very small frequencies or CLL-unrelated rearrangements. **, p=0.005.

After identifying all ‘truly-unmutated’ clonotypic IGHV-IGHD-IGHJ mu transcripts in each patient, we computed the number of the corresponding clonotypes as well as the number of the respective subclonal variants. In subset #1, the main CLL variant was present at a median frequency of 63.9% (range 56.2%-71.2%), whereas subclonal variants were identified in all cases (median: 22, range: 7-39 subclonal variants) accounting for a minor fraction of the respective repertoire (median frequency: 0.1%, range: 0.02%-2.27%). In subset #6, the main CLL variant was present at a median frequency of 54.0% (range: 41.1%-63.7%), while subclonal variants were again identified in all cases (median 7, range 1-10 subclonal variants) again accounting for a minor fraction of the total repertoire (median frequency: 0.1%, range: 0.03-0.21%). The remaining clonotypic background consisted of: (i) clonotypes unrelated to the main CLL clonotype, (ii) CLL-related subclonal variants with <100% IGHV identity, and (iii) subclonal variants of very small frequencies, which were discarded due to the high possibility of representing artefacts. (**Figure 1**).

### Evidence for SHM within the VH CDR3 in CLL cases assigned to stereotyped subset #1

Stereotyped subset #1 cases carry a 13 amino acid-long VH CDR3 with a highly conserved QWL motif at positions 4-6 encoded by the IGHD6-19 gene in reading frame 1. Our findings from the present NGS analysis were in line with this, since all subset #1 patients carried this QWL motif at the exact same positions. However, NGS also revealed subclonal variants in both this motif but also in other IGHV and IGHJ germline-encoded codons within the VH CDR3. The topological analysis of these variations revealed a preferential targeting of VH CDR3 amino acid positions 2, 4 and 12 versus the remaining codons (p<0.05).

**Figure 2:**
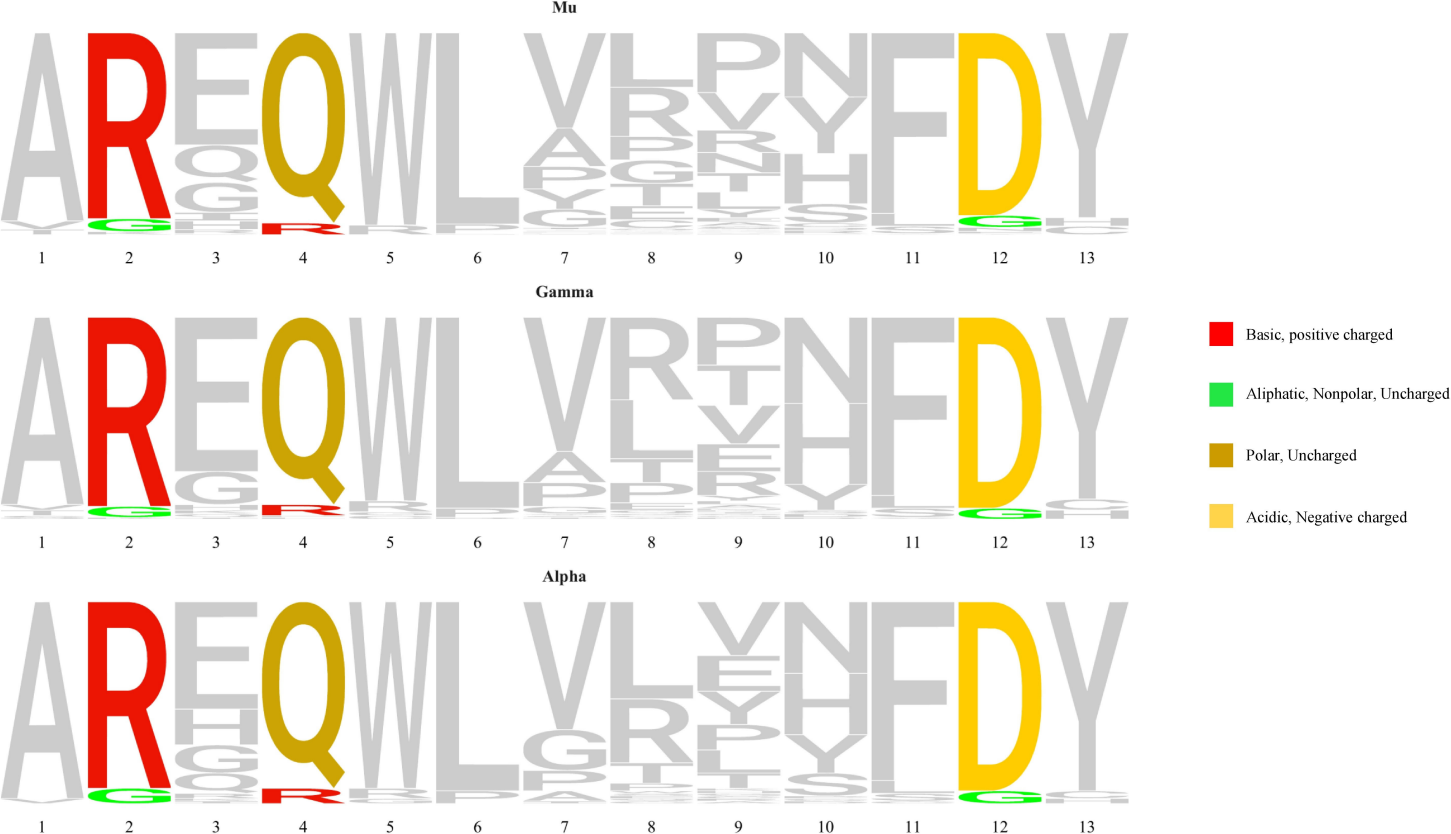
VH CDR3 amino acid sequence logos for subset #1 patients showing in color the positions with the most frequent substitutions in the VH CDR3 of the dominant mu and the switched gamma or alpha variants, as well as the most frequent substitutions at these positions. Each amino acid is colored according to its physicochemical properties, using the IMGT coloring system (https://www.imgt.org/IMGTeducation/), namely the basic (red) arginine as well as the acidic (yellow) aspartic residues mutate to the nonpolar (green) uncharged glycine residue, whereas the uncharged and polar (dark yellow) glutamine is replaced by a basic arginine residue (red).

In more detail: (i) all 12 patients carried a subclonal R>G aa substitution at position 2 of the VH CDR3 (IGHV-encoded; median frequency: 0.12%, range: 0.08-0.13%); (ii) 11/12 patients (91.6%) carried a subclonal Q>R aa substitution at position 4 within the QWL motif (IGHD-encoded; median frequency: 0.1%, range: 0.09-0.12%); and, finally, (iii) all 12 patients carried a subclonal D>G aa substitution at position 12 (IGHJ-encoded; median frequency: 0.11%, range: 0.08-0.13%). Notably, all three aforementioned subclonal substitutions resulted from an a>g nucleotide transition at the respective codons, with the resulting amino acid changes conferring different physicochemical properties from the germline-encoded ones (**Figure 2**).

Next, in order to further characterize recurrent SHMs identified within the VH CDR3 of subset #1 cases we investigated whether these SHMs were also present in switched gamma and alpha clonotypic variants. The main switched variants were 100% identical to the main mu variant in all cases. To ensure that this finding reflects actual immunogenetic relatedness, the respective primer sets used for the amplification of gene rearrangements of different isotypes were different, while the respective PCR amplicons were prepared and sequenced in different batches. Indeed, 8/12 (75%) patients shared the R>G aa substitution at VH CDR3 position 2 in either the gamma or the alpha switched variants or both (median frequency: 0.11% for both transcripts, range: 0.05-1.08%, median frequency for gamma and alpha trasncripts: 0.08 and 0.14%, respectively). Moreover, the Q>R aa substitution at VH CDR3 position 4 was shared by 8/12 (75%) patients in at least one gamma (median frequency: 0.08%, range: 0.05%-0.1%) or alpha switched variant (median frequency: 0.09%, range: 0.05-0.13%). Finally, the D>G aa substitution at VH CDR3 position 12 was shared by 7/12 (66%) patients in at least one gamma (median frequency: 0.1%, range: 0.09-0.13%) or alpha switched variant (median frequency: 0.09%, range: 0.05-0.12%). Cases where we did not document switched variants with the aforementioned replacement SHMs exhibited overall lower levels of VH CDR3-derived subclonal heterogeneity in the switched variants compared to the main, mu-expressing CLL variant (median 22 subclonal variants for the main variant vs. median 15 and 8 subclonal variants for gamma, alpha switched variants respectively); this explains, at least in part, the absence of VH CDR3-focused SHM in some of these cases.

### Evidence for SHM within the VH CDR3 in CLL cases assigned to stereotyped subset #6

Stereotyped subset #6 is characterized by the expression of a 21 amino acid-long VH CDR3 with high conservation regarding not only the IGHV-, IGHD- or IGHJ-encoded codons but also the IGHV-IGHD and IGHD-IGHJ gene junctions. However, VH CDR3 codon 9, which is IGHD-encoded, has been reported to display variation (12): indeed, a significant fraction of cases analyzed by Sanger sequencing bear an Isoleucine (I) residue rather than the Valine (V) residue encoded by the germline sequence of the IGHD3-16 gene in reading frame 2.

**Figure 3:**
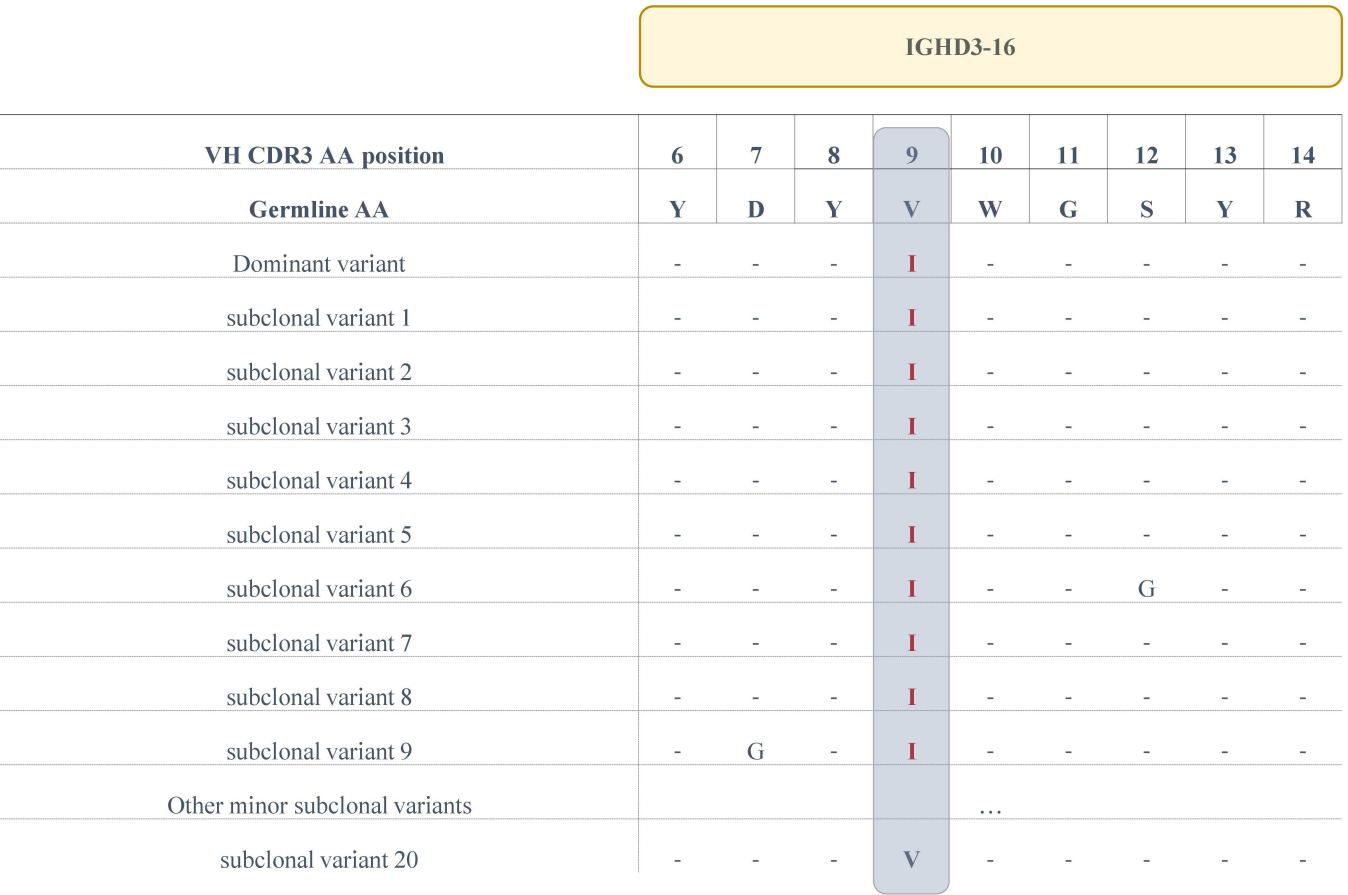
Alignment of the IGHD3-16-encoded region of the VH CDR3 of the main clonotype and subclonal variants for patient H73. The sequences of all variants (at the amino acid level) that display a V>I aa substitution at position 9 are depicted, as well as a single subclonal variant bearing the germline IGHD3-16-encoded Valine at the same position.

In the present series, 2/4 (50%) subset #6 patients were known by previous Sanger sequencing to carry a clonal V>I aa substitution at VH CDR3 codon 9. Results from the current NGS experiments confirmed this finding; in more specific, ‘truly unmutated’ clonotypic rearrangement sequences bearing this change represented 64.7% of the total repertoire of patient H73 and 41.2% of patient H74, respectively. The V>I aa substitution was also identified in all subclonal variants of these patients with a median frequency of 0.1% (range: 0.08%-0.21%). Unlike subset #1, switched variants are not detected in subset #6 (19), therefore we could not assess recurrence of the documented V>I aa substitution in a similar manner to that of subset #1. That notwithstanding, it is worth noting that we detected a minor variant (33 reads) carrying the germline-encoded V residue at codon 9 in patient H73 (**Figure 3**), which we report despite being below the adopted cutoff of 66 reads on the grounds that V is the germline residue. On this evidence, it becomes apparent that the clonal V>I substitution in patient 73 represents a true SHM result rather than a sequencing artefact.

Similar to subset #1, NGS analysis disclosed additional recurrent SHMs at the subclonal level, clustered at particular VH CDR3 codons. In more detail, 3/4 (75%) subset #6 patients carried a R>G aa substitution at VH CDR3 position 2 (IGHV-encoded; median frequency: 0.11%, range: 0.09%-0.13%), whereas all 4 patients (100%) carried a F>V aa substitution resulting from a t>g transversion at VH CDR3 codon 19 (IGHJ-encoded; median frequency: 0.2%, range: 0.13%-0.21%).

### SHM topology in relation to AID hotspots

In order to obtain more supportive evidence for the origin of the identified variations, we scanned the IGHV, IGHD, IGHJ germline nucleotide sequences of the VH CDR3 for the topological overall between the identified variants and hotspots of the activation-induced deaminase (AID). Starting from subset #1, all three mutations that were subclonally detected in the majority of patients resulted from a>g transitions. Such mutations usually arise after U:G mismatch repair *via* the mismatch repair machinery pathway (MMR) and are adjacent to the deaminated cytosine of the AID WRC/GYW hotspots (W=A/T, R=A/G, Y=T/C. Indeed, the 3’ IGHV-encoded arginine (R) residue, as well as the IGHJ4-encoded aspartic acid (D) residue were localized in close proximity with two of these hotspots, namely TGC/GCA in the IGHV gene, and AAC/GTT in the IGHJ gene. The Q>R aa substitution found within the IGHD6-19-encoded QWL motif and detected in 91.6% of subset #1 patients, also resulted from an a>g transition. Interestingly, in 9/11 patients in which a detected subclonal variant was found to bear the Q>R aa substitution, we observed generation of an overlapping WGCW SHM hotspot or a WRC motif (AGCA or AAC) either at the N1-IGHD junction, or directly at the IGHV-IGHD junction, adjacent to the codon encoding for the glutamine (Q) residue. These findings indicate that there may be selective pressure for SHM targeting at this particular position.

**Figure 4:**
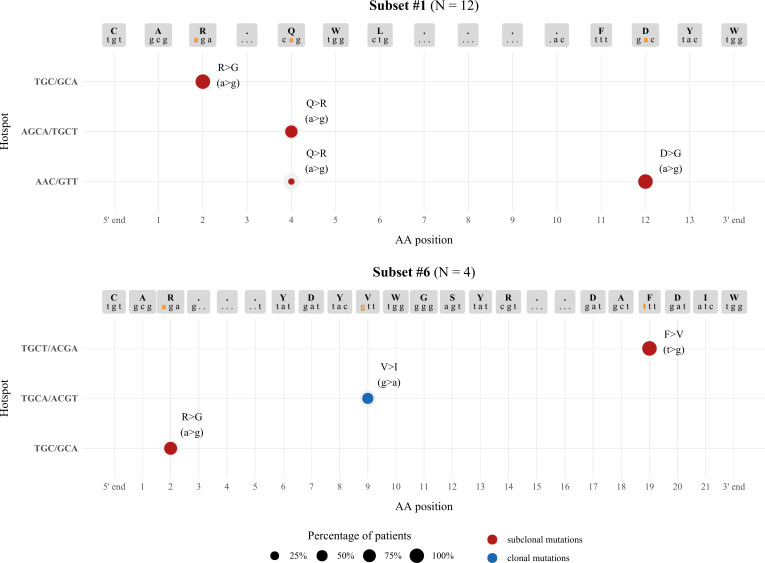
Topology of SHMs observed within the VH CDR3 in relation to AID hotspots. The size of each node represents the percentage of patients exhibiting a particular SHM, while the graph coordinates of each node correspond to the AID hotspot involved in each SHM. Different colors are used to distinguish between clonal (blue) and subclonal (red) SHMs.

For subset #6, the clonal V>I aa substitution results from a g>a transversion located within an overlapping motif present in both strands (AGCT/ACGT), likely generated after mismatch repair from the replication system (**Figure 4**).

Altogether, this analysis supports the notion that the mutations occuring within the VH CDR3 likely represent *bona fide* SHM rather than NGS artefacts.

## Discussion

Determination of the SHM status in CLL is key to disease prognostication and prediction of clinical outcome (20). Going beyond the binary distinction between U-CLL versus M-CLL, increasing evidence supports the theory that BcR IG stereotypy may assist in refining risk stratification, given that cases belonging to the same stereotypd subset share several clinical and biological features, including recurrent SHMs (12, 21–23).

The study of SHM in various B cell malignancies, including CLL, has offered valuable insight into disease ontogeny and evolution, particularly as it concerns derivation and interactions with antigens (24, 25). Particularly for U-CLL, which is the focus of the present work, the prevailing view is that U-CLL likely derives from a cell differentiating independently from a germinal center reaction, yet the cell(s) of origin remain to be conclusively defined (26–29). That notwithstanding, U-CLL is defined based on the 98% germline identity cutoff, thus it represents an assortment of cases with varying SHM status, ranging from limited to none, the latter referred to as ‘truly-unmutated’. This definition implies a complete absence of SHM, which may be misleading considering that the prognostically relevant determination of SHM status is confined to the sequence of the rearranged IGHV gene. Hence, the VH CDR3, i.e the most diverse part of the BcR IG and most relevant for antigen recognition, is completely ignored.

In the present work we sought to obtain evidence for the existence of SHM in IGHV-IGHD-IGHJ gene rearrangements from ‘truly unmutated’ CLL clones. To that end, we focused on U-CLL stereotyped subsets #1 and #6 and selected cases expressing IGHV genes with 100% identity to the respective germline IGHV gene, as previously documented by Sanger Sequencing. We applied NGS in order to obtain a comprehensive view of the (sub)clonal architecture of the BcR IG gene repertoire (30). Moreover, given our choice to study ‘truly unmutated’ rearrangements, we applied a series of stringent filters to our NGS data in order to ensure the absence of SHM in the VH FR1 to VH FR3 as well to exclude potential sequencing artefacts.

Intraclonal diversification analysis revealed the presence of subclonal branching in both subsets #1 and #6. This is in keeping with previous studies reporting the existence of subclonal heterogeneity within the clonotypic CLL BcR IG gene rearrangements likely in the context of ongoing antigen interactions (31–33). That said, the aforementioned studies focused on the IGHV-encoded regions (VH FR1 to VH FR3) and, moreover, examined mainly M-CLL cases. Hence, our results show not only that subclonal heterogeneity is present in U-CLL, but also that this diversification can be the result of specific SHM targeting of the VH CDR3, even if the clonotypic IGHV gene exhibits complete lack of SHM. The observed differences in the levels of subclonal branching between stereotyped subsets #1 and #6 are not entirely surprising since: (i) subset #6 is known for very high levels of conservation in terms of the aa composition of the VH CDR3 (12), which could be likely reflected at the subclonal level, and (ii) each stereotyped subset represents a distinct entity, with notable differences in the aa composition and, thus, structure of the BcR IG, arguably affected by distinct factors/stimuli driving (sub)clonal evolution (34–36).

In regard to stereotyped subset #1, we detected recurrent subclonal events within the IGHD-encoded QWL motif as well as in the IGHV- and IGHJ- encoded parts of the VH CDR3. Such recurrence combined with the presence of the same events in the respective switched variants strengthens the argument that these substitutions are indeed true SHM-induced mutational events, likely attributed to ongoing antigenic interactions post-transformation in subset #1. Turning to subset #6, both the F>V aa substitution subclonally detected in the IGHJ3-encoded FDIW motif of all patients, as well as the clonal V>I aa substitution observed in half the cases resulted in a conservative substitution that is not anticipated to affect significantly the overall physicochemical properties of the respective regions, maintaining their nonpolar profile.

Similar to normal B cells (37), SHM in CLL preferentially clusters within certain hotspot motifs which represent targets of the AID enzyme (38). Of note, all VH CDR3 nucleotide changes identified in subset #1 as well as the respective changes in the subclonal variants of subset #6 patients were located at a/t sites adjacently to AID hotspots, consistent with a non-canonical SHM mechanism (39). These findings strengthen our hypothesis that the mutations reported in the present work are indeed results of the SHM process. The clonal V>I aa substitution in subset #6 resulted from a g>a transversion at the respective codon, which is located at an overlapping WGCW hotspot, pointing to canonical SHM as the underlying mechanism.

Overall, the herein reported VH CDR3-focused SHM, present in all patients of our cohort, may imply that U-CLL BcR IG may diversify post-transformation through ongoing antigen interactions critically mediated *via* the VH CDR3 region. These results question the existence of ‘truly-unmutated’ CLL, with important ontogenetic implications. Their clinical relevance cannot be determined presently, especially given the limited cohort size, and will require formal investigation in large, well characterized clinical cohorts.

## Data availability statement

The datasets presented in this study can be found in online repositories. The names of the repository/repositories and accession number(s) can be found below: The NGS data generated for this study have been deposited and are publicly available in the European Nucleotide Archive (ENA of EMBL-EBI) under accession number PRJEB56468 (ERP141418).

## Ethics statement

The studies involving human participants were reviewed and approved by The Bioethics committees of Centre for Research and Technology, Hellas and Aristotle University of Thessaloniki. The patients/participants provided their written informed consent to participate in this study.

## Author contributions

ES performed experiments, analyzed data and wrote the manuscript. LZ-I, NP, FP assisted with bioinformatics analysis and data visualization. GK assisted with statistical analysis. SN assisted with experiments. NS provided clinical samples. EG, AA, FD, AC assisted in the interpretation of results. KS designed and supervised the study and wrote the manuscript. All authors contributed to the article and approved the submitted version.
